# High ANC coverage and low skilled attendance in a rural Tanzanian district: a case for implementing a birth plan intervention

**DOI:** 10.1186/1471-2393-10-13

**Published:** 2010-03-19

**Authors:** Moke Magoma, Jennifer Requejo, Oona MR Campbell, Simon Cousens, Veronique Filippi

**Affiliations:** 1 Infectious Diseases Epidemiology Unit, Department of Epidemiology and Population, Health, London School of Hygiene and Tropical Medicine, Keppel Street, London WC1E 7HT, UK; 2 Department of Reproductive Health and Research, World Health Organization, Geneva, Switzerland

## Abstract

**Background:**

In Tanzania, more than 90% of all pregnant women attend antenatal care at least once and approximately 62% four times or more, yet less than five in ten receive skilled delivery care at available health units. We conducted a qualitative study in Ngorongoro district, Northern Tanzania, in order to gain an understanding of the health systems and socio-cultural factors underlying this divergent pattern of high use of antenatal services and low use of skilled delivery care. Specifically, the study examined beliefs and behaviors related to antenatal, labor, delivery and postnatal care among the Maasai and Watemi ethnic groups. The perspectives of health care providers and traditional birth attendants on childbirth and the factors determining where women deliver were also investigated.

**Methods:**

Twelve key informant interviews and fifteen focus group discussions were held with Maasai and Watemi women, traditional birth attendants, health care providers, and community members. Principles of the grounded theory approach were used to elicit and assess the various perspectives of each group of participants interviewed.

**Results:**

The Maasai and Watemi women's preferences for a home birth and lack of planning for delivery are reinforced by the failure of health care providers to consistently communicate the importance of skilled delivery and immediate post-partum care for all women during routine antenatal visits. Husbands typically serve as gatekeepers of women's reproductive health in the two groups - including decisions about where they will deliver- yet they are rarely encouraged to attend antenatal sessions. While husbands are encouraged to participate in programs to prevent maternal-to-child transmission of HIV, messages about the importance of skilled delivery care for all women are not given emphasis.

**Conclusions:**

Increasing coverage of skilled delivery care and achieving the full implementation of Tanzania's Focused Antenatal Care Package in Ngorongoro depends upon improved training and monitoring of health care providers, and greater family participation in antenatal care visits.

## Background

Three core health sector strategies are identified within the maternal health community as critical for reducing maternal and early neonatal deaths. These include comprehensive reproductive health care; skilled care for all pregnant women, especially during delivery; and emergency obstetric care for all women and infants with life-threatening complications [1]. Political commitment to maternal and newborn health has historically been low, however, and most women in developing countries do not receive these recommended aspects of care. Maternal mortality remains high in the developing world and contrasts sharply with the low levels of maternal mortality in developed countries. Comparison of the lifetime risk of maternal death in industrialized countries (1 in 8000) versus in Sub-Saharan Africa (1 in 22) and Asia (1 in 59) presents a particularly stark picture of persisting global disparities in maternal health [2]. Addressing these inequities by increasing women's access to reproductive health services-including skilled and emergency care at delivery-must be a global priority, particularly if the world is to achieve Millennium Development Goal 5 (MDG 5) (improve maternal health).

Antenatal care (ANC) visits constitute one of the few times women in many resource-poor settings seek care for their own health [3], and, represent an important opportunity to help women best prepare for birth, as well as inform them about pregnancy-related complications, and the advantages of skilled delivery care[4,5]. Several studies show that women who attend ANC are more likely to seek skilled delivery care [6-9]. Nevertheless, at least 20% of all women who attend ANC four times or more in sub-Saharan African and in Asian countries do not seek skilled delivery attendance [8]. Efforts to understand the complex factors underlying this discrepant pattern between high utilization of antenatal care and low use of skilled birth attendance in these two regions where over 85% of all maternal deaths occur must be undertaken [10,11]. These efforts should include investigation into how antenatal care services can be used to promote skilled delivery care and effective management of obstetric complications.

Three delays contribute to the high maternal mortality in developing countries: delay in seeking care, delay in reaching care and delay in receiving care [12]). Overcoming these delays requires interventions at both the supply and demand sides of health provision [13]. Birth preparedness and complication readiness are promoted to reduce these delays so that women with pregnancy-related life threatening complication receive appropriate care promptly [14]. Although the risk-based approach in ANC has not been proven reliable in predicting women likely to develop life threatening conditions, pregnant women are motivated to decrease the risk to their unborn babies [15].

Tanzania is a sub-Saharan African country characterized by very high maternal mortality (MMR is 578 per 100,000 live births), high coverage of ANC (over 90% attend at least one ANC visit and approximately 62% attend four or more visits), and low coverage of skilled delivery and immediate postpartum care (43% and 13% respectively) [16]. At the time of this study (2007 and 2008), antenatal, delivery and post-natal care in Tanzania were free or highly subsidized. Tanzania's Focused Antenatal Care Package based on the World Health Organization's (WHO) new ANC model was introduced in 2002 [17]. Recent evidence showing that the orientation package is available in only one third of health units indicates slow progress in the implementation of the new package [18]. An important element of this package is counselling on birth preparedness and complication readiness. However, an observational time-motion study in Tanzania found that the average time spent on counselling during ANC visits falls far short of estimated time requirements to cover essential topics [19]. Thus, it appears ANC is currently underutilized as an opportunity to encourage health seeking of skilled delivery care.

A key aim of this exploratory study was to gain an understanding of the socio-cultural and health systems factors influencing women's decisions to seek antenatal, skilled delivery and immediate post-partum care in Ngorongoro, a rural district in Northern Tanzania. Examining the barriers and facilitating factors women experience to accessing these reproductive health services is an important first step towards identifying appropriate interventions to introduce in the study area to increase use of skilled delivery and immediate post-partum care. Maternal health conditions in Ngorongoro reflect the national pattern. The MMR among antenatal attendees is estimated at 642 maternal deaths per 100,000 live births (CI 329;955) [Johnson et al, 2005 unpublished-with permission]. Antenatal clinic attendance in Ngorongoro is over 90% while only 7% of women receive skilled delivery care. Coverage of postnatal care six to eight weeks after delivery is almost universal but receipt of immediate postnatal care is rare. The Focused Antenatal Care Package has not been widely implemented.

## Methods

### Study site

The study was conducted from October 2007 to May 2008 in Ngorongoro, one of six districts in the Arusha region, Northern Tanzania. Ngorongoro has 21 health units (two hospitals, 19 dispensaries) [Ngorongoro district annual health report, 2005-with permission]. All except six (the two hospitals and four dispensaries) are government owned. The two hospitals offer comprehensive emergency obstetric care while the dispensaries offer basic emergency obstetric care. The hospitals operate an extensive network of mobile health clinics to deliver services to remote areas. In addition, they have maternal waiting wings where women from remote villages can stay prior to labour onset. Since very few women deliver at these hospitals, these wings are rarely used. The dispensaries are connected to the hospitals and district health headquarters by radio communication, although mobile phones are replacing the radio linkages. In emergencies, patients are transported to the hospitals by ambulances or plane for free or at nominal cost. Nine health units were selected for the study (both hospitals and seven randomly selected dispensaries). Study participants were selected from these units and their catchment areas. Antenatal, delivery, postnatal and care for children under-five years of age were free at all selected units except one dispensary where women paid approximately 2US$ for delivery care.

Ngorongoro includes three administrative divisions - Ngorongoro, Loliondo, and Salei - composed of wards with approximately 6 villages each. Ngorongoro and Loliondo are populated predominately by Maasai (80% of the total district population) and the Watemi ethnic group (18% of the total district population) occupy part of Salei. The Maasai are primarily pastoralists and the Watemi are small scale agriculturalists. According to the 2002 Tanzania National Census, the population of Ngorongoro was 129,776 of which 29,499 were women in the reproductive age group [20]. Using the national crude birth rate of 44.8 per 1,000 population for rural Tanzania [16] and the district population estimate of at least 140,625 in 2006 (personal communication with district planning department), about 6,300 deliveries occur in the district each year.

### Sample Selection

Study participants were purposively selected to ensure adequate representation of the Maasai and Watemi ethnic groups and all key stakeholders in maternal health in Ngorongoro district. This sampling strategy enabled us to explore women's decision making process about where to deliver, and the perspectives on pregnancy and childbirth of health care providers and Maasai and Watemi community members. Examining the perspective of health care providers was of particular importance because the culture of biomedicine has been recognized as key to the transmission of stigma, the incorporation of racial bias in institutions, and the entrenchment of health disparities across minority groups [21].

The Maasai and Watemi in Ngorongoro are tightly knit communities and speaking about sensitive issues with strangers may not elicit detailed responses (MM personal experience). Thus, people acquainted with one another were recruited for the FGDs as recommended under such circumstances [22]

Users of antenatal, delivery, and post-delivery care were invited to participate in focus group discussions (FGD) during unannounced visits to the MCH clinics. Six were women who had previously delivered in health units. Lists of all practicing Maasai TBAs in the surrounding catchment areas were obtained from the health units. The 36 total TBAs invited to participate in the FGDs were divided into three groups (one group of 8 corresponding to the areas served by the dispensaries, and two groups of 12 and 16 serving the two hospitals). Watemi TBAs play a very limited role in antenatal, labour and delivery care and were purposely excluded. MM (principle investigator and previously was a practicing obstetrician in the area) obtained lists of all elected elders from the ward offices. A maximum of 16 Elders from the wards in each of the three divisions were invited to participate in the FGDs.

The key informant interview participants included 2 women with previous experience with antenatal and delivery care services in the district who were recruited during a hospital outreach visit, their husbands, 2 women admitted to the hospitals (one for pregnancy related complications and another for delivery related complications), 2 traditional leaders, the district MCH coordinator, one prominent Maasai TBA, and senior Elder males from the two ethnic communities (2). All key informants were interviewed once except the TBA and the district MCH coordinator who were interviewed twice to clarify discrepancies that surfaced during data collection.

### Data collection

Data were collected through focus group discussions (FGDs), key informant interviews, and participant observation. MM observed and participated in the provision of care provided at antenatal clinics (six clinic days observed) and in the labour and delivery rooms of the two hospitals and one dispensary (four deliveries observed). All observations were recorded on the same day in a field diary.

15 FGDs were conducted involving a total of 160 participants: three FGDs with care providers at the health units (total of 18 providers); six FGDs with women seeking antenatal, delivery and post-delivery care at these health units (total of 66 women); three FGDs with TBAs (total of 36 TBAs); and three FGDs with Elders (total 40 men). All care providers except three were trained to the level of nurse midwife and 17 of the 18 were women.

A group of 6-16 participants formed a single focus group. FGDs were conducted by trained Maasai and Watemi facilitators in the Ma, Kitemi, or Kiswahili languages. The discussions were audio-taped using a digital voice recorder and a trained sociologist noted all non-verbal communications. Separate FGDs for men and women were held under a tree at sites chosen by the participants, emulating communal gatherings in the two ethnic groups. Holding discussions outside the health units also enabled participants to speak more freely about their perceptions of available professional care. FGDs with care providers were held at health units.

MM conducted the key informant interviews in Ma, Kitemi or Kiswahili. Semi-structured interview guides were used for both FGDs and key informant interviews and were initially pre-tested for cultural relevance in the communities surrounding the participating health units. This approach allowed us to guide the discussions around key topics related to pregnancy and childbirth without imposing a set of beliefs on participants. The main topics covered included reasons for seeking antenatal, delivery and post natal care; the decision-making process for accessing antenatal, skilled delivery and postnatal care; perceptions of the quality of antenatal, delivery and postnatal care at available health units; perspectives on planning for birth and emergencies; and the factors underlying the pattern of almost universal coverage of antenatal care services but low utilization of skilled delivery care. Relevant issues surrounding the uptake of Focused Antenatal Care and Prevention of Mother-To-Child Transmission (PMTCT) programs in the district were also explored.

### Data analysis

All FGDs and key informant interviews were transcribed and translated into English by two investigators fluent in Ma, English, Kitemi, and Kiswahili. Where there was no consensus, the investigators reviewed the transcriptions and original recordings until agreement was reached. The transcripts were entered into Microsoft Word and coded using principles of the grounded theory approach [23]. Specifically, the coding process involved identifying major themes in each of the transcripts. During data analysis, identified themes were compared across the transcripts to determine differences and similarities in the perspectives of the study participants on childbirth and the factors influencing women's decisions to seek skilled delivery care. The focus group discussions provided us with information on normative views on childbirth and care seeking behaviours operative in the two ethnic communities and among health care providers. The key informant interviews complemented this information by allowing us to explore in more detail individual experiences with reproductive health care services in Ngorongoro.

The process of triangulation was used to validate the findings and involved comparing the identified themes from the FGDs and key informant interview transcripts with the participant observations [24]. Discrepant findings between the observations and the transcripts were addressed by follow-up informal discussions with care providers.

Ethical approval was obtained from the Institutional Review Boards of the World Health Organization, London School of Hygiene and Tropical Medicine, and the National Institute for Medical Research in Tanzania. Permission was also granted from Arusha region and Ngorongoro district administrative and health authorities, staff at participating health units, and traditional leaders in Ngorongoro district. Informed consent was obtained from all participants.

## Results

The results are organized into four dimensions of obstetric care: antenatal, labour and delivery, emergency, and postnatal care. Within each category, major themes related to the factors influencing women's ability to access care and community perceptions of the quality of available care are presented. Health care provider perspectives on these issues and key findings on PMTCT programs are also shown.

### Antenatal Care

#### Perceptions of the need for professional care and quality of available care

Antenatal care is highly regarded in both communities, the quality of available care is considered good and most women feel obligated to attend. Regular ANC attendance is believed to guarantee healthier pregnancies and uneventful deliveries, and women who miss visits are considered at risk of poor pregnancy outcomes. Reasons given for high ANC attendance include: perceived health benefits to women and their unborn babies from receiving periodic examinations, vaccinations, and treatment for detected diseases; reassurance of the pregnant woman's wellbeing; referral of women with problems to hospitals for needed care; receipt of antenatal clinic attendance cards that guarantee free health care; and assistance with transport to reach health units for delivery.

Care providers interviewed reported that the ANC services they deliver meet the needs of most of their patients, and that they fully inform women of the benefits the care offers. Providers noted that effective treatment of diseases such as syphilis and the provision of preventive drugs for anaemia and malaria convinces women of the value of ANC, and fosters a sense of trust which encourages them to return for additional visits. Occasional drug and vaccine stock-outs, language barriers preventing effective communication with patients, poor communication with the district health headquarters, and the remoteness of women's residences from health units were mentioned as factors impeding their ability to consistently deliver quality ANC services.

*"Most women come to ANC clinics because they have confidence in the benefits of the services. For example, women know the benefits of various vaccinations and will demand them even when it is not appropriate to receive them. It is not uncommon to see a woman complaining why she has not been vaccinated against tetanus even when her gestational age does not qualify her. They also like the drugs given during antenatal clinic visits." *[Service Provider FGD]

*"Benefits of ANC are well- recognized in our community. For example, a woman who has been losing pregnancies may come to the clinic seeking assistance and after screening, a provider discovers that she has syphilis. If treatment is provided and she manages to carry successful subsequent pregnancies, she will believe in the services. Such women are many in Maasailand." *[Service Provider FGD]

### ANC and women's Empowerment

The women interviewed described ANC visits as beneficial for their health, and a rare opportunity to leave their households and exert control over their pregnancies. Although Maasai and Watemi women usually require permission from husbands to leave their households, they do not typically need permission from their husbands to attend ANC clinics. Only women married to older and relatively less educated husbands reported needing such permission.

*"This clinic has given us power. We meet and discuss many issues and our husbands are supportive. For example, recently the examination house was damaged and we met here on a non-clinic day and decided to contribute money to repair it. It was repaired in one week and now it is OK. If we have a serious problem we send the village health worker to the village elders' meeting and her opinions are honoured." *[Mtemi mother FGD]

*"No; we do not need permission from anyone. Most men are motivated to assist us to attend clinics. They want us all to attend." *[Maasai pregnant woman FGD]

### TBAs

The role of TBAs differs between the two ethnic groups. TBAs are an integral part of a Maasai woman's care during pregnancy, labour, and delivery. Maasai TBAs commonly accompany women to antenatal clinics, examine women at home, and refer them to health units for care if they identify a potential problem. TBAs in the Watemi community are only used in emergency situations when quick transport to health units is not possible during labour and delivery.

Health care providers expressed concerns that Maasai TBAs may dissuade women from delivering at health units for the following reasons: 1) Maasai women's faith in TBAs to refer them to health clinics when necessary if complications occur during labour or delivery, 2) Maasai women's practice of seeking TBA approval before heeding referrals, and 3) admission by some TBAs that they would lose status and the gifts they receive from attending deliveries if all women opt to deliver at health units. All key informants, TBAs and Elders interviewed explained that decision on place of delivery depends primarily on family (especially the husband's) preferences. They noted that TBAs are willing to encourage all women to deliver at health units.

### Labour and Delivery Care

Most women in Ngorongoro deliver at home assisted by TBAs (Maasai) or other female relatives and neighbours (Watemi). As soon as labour begins, women contact their TBA or female relatives who stay with them through labour and up to five days post-partum.

*"We are always there in labour up to a few days after delivery to take care of both the mothers and the babies and we decide on what they should eat and do" *[Maasai TBA-Key Informant]

Delivery care at health units is usually sought as a last resort after serious complications have developed. Barriers identified as detracting from women's ability to access skilled delivery and emergency obstetric care include: 1)distance from health units 2) lack of reliable and affordable transport 3) lack of advanced planning for accessing delivery care units 4)widely held beliefs that pregnancies labelled as 'normal' during ANC visits will result in successful deliveries at home 5) failure of providers to convey information about the importance of skilled delivery care for all women, and 6) women's low social status and inability to independently make labour and delivery decisions. These factors are discussed in more detail below.

### Barriers to access skilled delivery care - planning in advance, transportation and cost issues

The women, husbands, TBAs, and Elders interviewed agreed that the largest obstacle to receiving skilled and emergency obstetric care is failure to plan in advance for transport. Planning in advance for delivery is not part of traditional practice in the two communities where home delivery is the norm. For most Maasai and other women living in remote villages, transport to health units for delivery or emergency obstetrical care is unreliable and unaffordable. Transport costs are not as prohibitive for Watemi women who have more financial security.

*"Home delivery occurs because we do not plan for transport to the nearby health units in advance. I believe if we can plan in advance, we will be able to travel to health units before the onset of labour pain (uchungu.)." *[Recently delivered Mtemi FGD]

*"Transport costs are not much unless you hire a car in case of emergency. The usual cost is five thousand shillings (~4USD). This is less than the price of a tin of beans *(5 L tin). *We all have farms and delivery at health units is free." *[Mtemi woman FGD]

The interviews showed that most community members are aware that obstetric and child care services are free or highly subsidized. Thus, perceived costs of services are not likely a major barrier to seeking skilled delivery care in the study area.

"*It is well known that health unit delivery at Wasso hospital is free and at Digodigo health centre we pay a token of three thousand shillings (~USD 2.5) for everything. Money is not a problem anymore; thanks to our government." *[Mtemi woman FGD ]

The discussions with community members and observations of care provided at the ANC clinics showed that health care providers are not consistently counselling women on the importance of making transport arrangements and planning in advance for delivery. One specific communication problem mentioned was providers' failure to inform women about the meaning of the expected date of delivery. Women in the two communities are interpreting the date listed on their antenatal cards as their actual delivery date and are waiting until this time to make delivery plans. As a result, women who start labour before their expected delivery date often end up delivering at home even if they expressed interest in delivering in health care facilities.

### Barriers to seeking skilled delivery care - Social roles

The Elders and TBAs explained that children in both ethnic groups are cared for by other relatives or neighbours in the mother's absence, and co-wives are expected to assist each other when necessary. These supportive practices, however, do not offset women's perceptions of the opportunity costs of delivering at health units. The need to resume household responsibilities soon after delivery was mentioned as a key reason women opt to deliver at home.

*"We *[women]*work too hard to care for our families even when we are pregnant to think of leaving our houses unattended to seek delivery care at health units." *[pregnant Mtemi FGD]

*"You know what, Watemi women are the workforces for their husbands. We look after cattle and farms. It can happen that you start labour when you're away from home taking care of your husband's cattle and end up delivering in the bush. When you come back home with the baby, this is seen as normal and there is usually nobody to assist you with household chores unless someone from your home comes to assist." *[pregnant Mtemi FGD]

Husbands typically decide on place of delivery in both ethnic communities, although the expressed preferences of Watemi women are beginning to be respected. Most Maasai women will only leave their households during labour after being granted permission by their husbands or developing a serious complication.

*"It is our decision as women. Men may have a say but finally our preferences are respected." *[Mtemi FGD]

*"How can you leave your household and you go to a health unit to deliver without your husband's consent? He is the one who pays for the costs." *[Maasai FGD]

Health care providers interviewed noted that Maasai and Watemi families will not readily accept the need for skilled delivery care without the approval of traditional leaders.

*"...traditional leaders are very powerful in this district such that whatever they say is automatically binding. Unless they are involved in convincing the community that health unit delivery is good, we will not succeed in having more women come to deliver at health units." *[Service provider]

### The normal and the natural: Who should deliver in health units?

Perceptions about the 'naturalness' and safety of home delivery is an obstacle to convincing women in the two ethnic groups of the importance of skilled delivery care in all cases. Although the women, TBAs and Elders from both communities expressed awareness of the potential risks of delivering at home, they stressed that delivering at health units is beneficial only for women with known complications. Women with "normal" pregnancies -- defined by the women participants as those with no problems or risk factors identified at ANC visits - are expected to be able to deliver without incident at home.

*"Most women do not have problems so they prefer to deliver at home. The few women with problems try to deliver at health units"*. [Maasai pregnant woman FGD]

*"Most women deliver at home because we are Maasai. Our mothers did so before us. We're just doing what others did." *[Maasai pregnant woman FGD]

Providers interviewed agreed that most women attend ANC visits for reassurance that their pregnancies are "normal" so that they can deliver at home. Providers re-enforce this pattern of behaviour by advising only pregnant women with identified risks to deliver at health units. The information that all pregnancies carry risks and that labour complications are often unpredictable is inconsistently communicated to women during routine ANC. The providers interviewed cited heavy workloads as the main reason for insufficient dialogue on the importance of skilled delivery care.

*"If health unit delivery was that much important, why is it that our providers fail to tell us so? We attend ANC well but they do not tell us. I'm eight months pregnant and I've attended ANC three times so far. Nobody has talked to me to deliver at any health unit." *[Pregnant Maasai FGD]

*"If indeed we're risking our lives with home delivery, I've a complaint to make; why is it that our providers fail to tell us this information? We hardly have time for thorough discussion on such issues. TBAs, on the other hand tell us to go to health units when problems have already developed. It may be too late for some." *[Recently delivered Maasai FGD]

A minority of women in the focus group discussions commented that problems experienced during labour and delivery among women with normal pregnancy histories would occur equally to those delivering at home or at health units. They claimed that only 'God' can protect women from a maternal death. These sentiments are rooted in traditional beliefs in both communities about pregnancy complications and maternal death as punishment for past transgressions:

*"In the past, serious and life threatening delivery complications were blamed on women's marital infidelities before or during pregnancy and complications were thought to be curses from elders and thus divine punishments upon women. Unless such women confessed to adulterous relationships to their delivery attendants, it was believed that they would die together with their attendants. All women who died during childbirth were believed to have committed adultery and brought shame to their families. To make sure that such secrets were not confided to other people, more and more women delivered with assistance from their own mothers to whom they would make confessions, and even when both had to die, it was seen as more preferable than dying with TBAs who were often of no blood relationship to the pregnant women, and thus confining TBAs to a lesser role. Over the years, the role of mothers have declined too because many women who develop problems and send to hospitals survive, the curses notwithstanding." *[Mtemi elder FGD]

### Communication barriers to seeking skilled delivery care

Community members interpret instances when health care providers at dispensaries fail to manage difficult cases as proof that such units are not suitable places to seek delivery care. Consequently, some participants mentioned that they would advise women to bypass nearby dispensaries and go directly to hospitals.

*"You know we do not trust our dispensary because they *[providers] *often fail to manage some conditions and refer them to the district hospital. Most of us would like to deliver at the district hospital where all conditions can be managed." *[pregnant Mtemi FGD]

The majority of women interviewed expressed concerns about specific routine and life saving procedures conducted by health care providers during labour, delivery and immediately postpartum. Maasai women perceive digital vaginal examinations performed at health units as painful, likely to damage the baby, and a cause of labour retraction. Some Watemi women and men described digital examinations performed by male providers as dehumanizing. In contrast, Maasai women felt that TBAs perform digital vaginal examinations gently and only when the baby's head is crowning. Maasai and Watemi participants explained that caesarean sections performed with no explanation provided in advance evoke fear in pregnant women that they will undergo unnecessary caesarean sections if they deliver in health units. Episiotomies and repairs of genital tears sustained during delivery also deter Maasai and Watemi women from seeking skilled delivery care. Genital tears are viewed in the two communities as inevitable complications of childbirth that do not require medical intervention. Health care providers interviewed blamed Maasai and Watemi women's low education level as the underlying cause of their misperceptions about routine labour and delivery procedures.

Squatting is the traditional labour position in the two ethnic groups. Skilled providers are only trained to assist women in the orthodox supine position. Study participants agreed that labour position is not a crucial factor in the decision making process about where women deliver. Some women felt that they should accept whatever position the provider decides is best. This view reflects the scope for deference to authority figures Maasai and Watemi women are expected to show:

"*It is bad for an expectant mother to be so demanding. How you deliver and who assists you does not matter but your safety and good care. Since not all providers are of our ethnic group, it is bad to impose our norms on others. We need to show respect to the providers as much as they respect us. If you have any birthing preferences and your provider suggests that she may not be comfortable with your choice, you must listen and abide. Even when we deliver at home, attendants may not like us to impose our choices on them. You must discuss and understand the best position to them in order to get the best out of their skills." *[Recently delivered Mtemi FGD]

Health care providers interviewed noted instances when they observed colleagues verbally abuse, and force Watemi and Maasai women in labour to bathe and put on hospital uniforms. MM also observed Maasai and Watemi women being told to bathe and put on uniforms by labour ward staff at the two hospitals.

### Perceptions of the quality of care

The health care providers and other reproductive health stakeholders interviewed all stated that TBAs and relatives are able to provide better emotional support and continuity of care in comparison to the type of care that is made available at the health units.

*"Not all units have qualified staff at the level of a midwife or above. It may be that clients feel safer with TBAs than such providers." *[Key informant]

TBAs and relatives are viewed by the Maasai and Watemi women as affordable (e.g., no transportation costs required), and able to meet their service expectations. These expectations include continual support and advice during pregnancy, delivery, and in the postpartum period, the provision of body massage throughout labour and delivery, and knowledge of a variety of delivery positions. The women do not think these services can be provided at health units where the sterile environment and languages used are foreign to them, and the few staff available have many other responsibilities in addition to labour monitoring. Nevertheless, women and Elder participants described health care providers at the clinics as well trained, competent, and compassionate. Most of the women interviewed who had previously delivered at health units hoped to have this opportunity in future pregnancies and would recommend delivering in health units to others. Of the women who had subsequent home births after delivering in health units, all expressed disappointment over being denied the opportunity to repeat this experience by their husbands.

*"I delivered my second child at Wasso hospital and the care was very good. I'm sure most women here have the same opinion *[most women nod to show that they agree with her]. *My husband sent me there because I had some problems. I was weak and my lower abdomen was painful. I tell you I'll never forget the courteous welcome and care I got. I vowed that my subsequent deliveries will all be at this hospital. My husband did not allow me to go there for delivery in my third pregnancy because I had no problem. I felt disappointed." *[pregnant Maasai woman FGD]

*"Most people know the good care provided at our health units especially at our two hospitals in the district. They have saved the lives of many women who would have died had it not been for the good care they received at these health units. We pray that they maintain the same level of care"*. [Maasai elder-Key informant]

*"Any sane person will never question the quality of care provided at the dispensary in our village and at the district hospital. They have saved many lives and our community is proud of having them. Most families and women speak highly of the care they provide"*. [Mtemi elder-Key informant]

TBAs interviewed described health care providers as highly trained and capable of managing normal deliveries as well as life threatening conditions arising during labour, delivery or the postpartum period. They listed health care providers' failure to encourage women with 'normal' pregnancies to deliver at health units as the key reason for the low utilization of skilled delivery care in the study area.

### Emergency Obstetric Care

In general, Maasai and Watemi women are able to leave their households to seek care at health units during emergencies without needing their husbands' permission. Maasai women in Loliondo, however, must receive permission from their husbands to seek skilled delivery care even under life threatening situations. In addition, advanced planning for possible blood donation in case women require a blood transfusion during and immediately after childbirth is not currently practiced nor encouraged during ANC visits.

### Postnatal Care

In 2004, the national guidelines for postnatal care were revised from advising women to seek care at six weeks postpartum to within one week, and at four and six weeks post delivery. Some health care providers interviewed were unaware of this policy change. Care providers rarely promote the importance of immediate post delivery care during routine ANC visits at the participating clinics (MM observation).

*"Providers tell us to come for after delivery care after one and a half or two months after delivery and not otherwise. Unless your baby is unwell, why should one attend earlier than this?" *[Mtemi pregnant woman-key informant]

*"The earliest time a woman should start to attend after delivery care at a clinic is one and a half months. If she fails, she must not do so later than two months. This is what providers tell us to teach women"*. [Maasai TBA- key informant]

The women participants explained that they attend post-natal clinics to be examined and treated for any post-delivery problems, to have their babies examined and vaccinated, and to receive under-five growth monitoring cards. The latter are required for obtaining birth certificates, free child health care, and to enrol children in primary schools. Watemi women and Elders interviewed stressed that not attending post-delivery clinics can be disastrous for both mother and baby as serious and life-threatening conditions can occur suddenly and unexpectedly after delivery. Maasai women believe that post-natal care is beneficial exclusively for the baby when a woman delivers normally.

### PMTCT

PMTCT services had been introduced at scale into routine antenatal care for less than one year at the time of the study. According to all participants, PMTCT services are widely supported in the Maasai and Watemi communities. PMTCT services such as HIV testing, counselling, and treatment are also prioritized in the study clinics and husbands are encouraged to participate in PMTCT sessions. Messages about the importance of skilled birth attendance are not relayed to couples during PMTCT counselling sessions.

Providers view maternal-to-child transmission of HIV as easily preventable through adequate counselling and the provision of antiretroviral therapy. PMTCT providers are expected to regularly submit performance reports to the funding organizations for HIV/AIDS care in Ngorongoro. In contrast, providers described maternal deaths as rare and not always preventable events often following unexpected complications. Supervision related to counselling on skilled delivery care falls under the auspices of the district supervision team and is much less rigorous.

### Suggested solutions for the low utilization of health units for delivery

Participants in FGDs and key informant interview made suggestions on how factors preventing women from utilizing health units in the district can be overcome. Notably, planning in advance for transport costs or moving near health units around the time of delivery, and change of providers' attitude and practices so that delivery under the assistance of care providers is consistently and widely promoted to all women during ANC visits were suggested. Men's involvement in ANC was mentioned as an opportunity to involve families and the wide community in maternal health issues. The need for maternal health stakeholders in the district to sensitize the community on maternal health issues so that there is a dialogue on norms and traditions for the low utilization of the available health units for delivery was also emphasized.

## Discussion

This study examines the perspectives of health care providers, Maasai and Watemi women, community members, and TBAs on antenatal, delivery, and post-natal care in Ngorongoro, Northern Tanzania. Examining and comparing these perspectives is an essential first step to identifying appropriate strategies for increasing women's utilization of skilled delivery and immediate postnatal care in the study setting. Understanding the factors influencing women's decision making process on where to deliver is also essential for determining how to improve antenatal care provider interactions with women and their families so that women are more likely to seek skilled delivery care, and have positive pregnancy experiences and birth outcomes.

The results show that for antenatal care to operate as a key intervention linking women to skilled delivery and immediate post-natal care in the study setting, health care providers need training on how to implement all aspects of the Focused Antenatal Care package and Tanzania's postnatal care policy. Specific areas identified that need to be addressed include: (1) insufficient counselling during ANC visits on the need for all women to receive skilled delivery and immediate post-natal care, (2) beliefs that only women with identified obstetric risk factors should be advised about skilled delivery care, (3) lack of encouragement of men/family members to participate in counselling sessions on the importance of skilled delivery care during ANC and PMTCT visits (4) lack of effective communication about routine and life saving procedures provided during labour, delivery and immediately postpartum (5) practices requiring women arriving in labour to bathe and put on hospital uniforms, and (6) lack of clear communication to community members on the referral structure and scope of services delivered at each level of care. Comprehensive training on these issues could improve the quality of the dialogue between women and health care providers at ANC clinics, and create greater motivation among women and their families to seek skilled delivery care in Ngorongoro [25,26].

Our findings are consistent with other research in Tanzania showing that time allotted for health education and counselling during ANC is minimal, with some women not receiving any counselling from their providers [20]. A recent study in a rural district in Tanzania suggests that only 25% of women at ANC clinics are informed of the danger signs in pregnancy and during delivery, and around 40% are informed of the danger signs after delivery [27]. Lack of adequate counselling and health education during routine antenatal care represents a missed opportunity to educate women, family members, and Elders about the importance of skilled delivery care for all women.

Fear of certain medical procedures during labour and delivery have been described in other Tanzanian and African settings as a major deterrent to women's willingness to seek skilled delivery care [28,29]. Concerns mentioned by community members about caesarean deliveries and repair of genital tears need to be more sensitively handled by providers to allay women's fears about such procedures and to explain why they are performed (e.g., repair of major cervical tears to prevent severe post partum haemorrhage).

Because pregnancy and childbirth are events charged with social meaning and often involve significant family and community participation, inclusion of family members in discussions about skilled delivery care during routine ANC is a strategy promoted in many developing countries [17,30-32]. Our study found that husbands and Elders in the Maasai and Watemi communities play key gate keeping roles in women's reproductive health, and are principally responsible for deciding where women deliver. However, husbands are not encouraged to participate in routine ANC and do not receive messages about safe delivery during PMTCT sessions.

Provider attitudes have been described in other African settings as an obstacle to increasing the utilization of skilled delivery care [33-35]. Observed and reported practices in the study setting such as requiring labouring women to bathe and put on hospital uniforms likely detracts from women's willingness to utilize skilled birth attendants. Recent evidence from Western Tanzania shows that improvements in provider attitudes towards their women patients and the availability of drugs and medical equipment resulted in a two-fold increase (from 43% to 88%) in women's preference to use the available health units for delivery [36].

The explanations given by providers for placing greater importance on PMTCT programs than on promotion of skilled delivery care during routine ANC have health systems implications. PMTCT programs in Tanzania are largely supported by international donors, are well monitored, and providers are highly motivated to comply with all reporting requirements. In contrast, monitoring of ANC and skilled delivery care is weak. Future studies should explore the role of supervision and support on effective implementation of the Focused Antenatal Care package in Ngorongoro and the performance of skilled birth attendants. Other studies should examine how the PMTCT component on the promotion of skilled delivery care can be better implemented.

Although MCH services at all study units except one were free, women experience many barriers to accessing skilled delivery care including distance from health units, transport difficulties, lack of autonomy to decide where they want to deliver, perceptions that home delivery is safe for 'normal' pregnancies, and traditional beliefs about the advantages of home births. Health care providers need to be aware of these issues and discuss them with women during ANC visits, particularly when they are counselling women on preparing for childbirth.

Our study has some limitations worth mentioning. Some of the original meanings might have been lost during translation and transcription. The use of two bilingual people during the translation and transcription processes minimized this problem. Recruitment of service users exiting clinics could have biased responses due to their desire not to alienate providers. Observation of care providers during ANC consultations and in labour rooms could have modified their behaviours, introducing bias in the observational results. To minimize the introduction of bias, the data collection methods were triangulated, and the information collected was found to be generally consistent across the methods. The number of participants with experience delivering in the health units was small (6), and their perceptions are likely to be underrepresented in our findings. The views of pregnant women who did not utilize antenatal care and their family members were not purposely targeted. Understanding why these women did not seek antenatal care could have added more insights into the reasons women in the study area do not utilize available health units for MCH care.

## Conclusions

We undertook this study as a first step towards understanding the factors underlying the divergent pattern between high ANC utilization rates and low usage of SBA in Ngorongoro, Tanzania. We used qualitative methods to examine the perspectives of health care providers, Maasai and Watemi women, community members, and other reproductive health stakeholders on antenatal, skilled delivery and postnatal care. Our findings show that ANC as currently provided in the study clinics represents a missed opportunity to effectively inform all pregnant women about the importance of receiving skilled delivery and immediate postnatal care. The quality of ANC services in Ngorongoro can be improved through training health care providers on implementing all components of the Focused Antenatal Care Model, Tanzania's postnatal policies, and on how to better communicate with their women clients, including their families. Further studies should be undertaken to validate our findings, including investigation into how community members came to view ANC services as essential to a healthy pregnancy, and the types of strategies needed to simultaneously improve the quality of available antenatal and skilled delivery care as well as demand for such services.

## Competing interests

The authors declare that they have no competing interests.

## Authors' contributions

MM designed the study, collected and analyzed the data, drafted the initial manuscript and reviewed subsequent drafts. VF, OMRC and SC participated in designing the study and reviewing the initial and final manuscripts. JR participated designing the study, developing the data collection tools, data analysis and reviewing the draft manuscripts at all stages. All authors approved the final version of the manuscript.

## Pre-publication history

The pre-publication history for this paper can be accessed here:

http://www.biomedcentral.com/1471-2393/10/13/prepub

## References

[B1] Starrs A Delivering for Women Lancet 2007 370 9595 1285 1287 10.1016/S0140-6736(07)61549-9 17933629

[B2] United Nations Children's Fund State of the world's children, 2008: Child survival 2007 New York. United Nations Children's Fund (UNICEF)

[B3] Carroli G Rooney C Villar J How effective is antenatal care in preventing maternal mortality and serious morbidity? An overview of the evidence Paediatr & Perinat Epidemiol 2001 15 suppl 1 1 42 10.1046/j.1365-3016.2001.0150s1001.x 11243499

[B4] Lindmark G Berendes H Meirik O Antenatal care in developed countries Paediatr & Perinat Epidemiol 1998 12 suppl 2 4 6 10.1046/j.1365-3016.12.s2.5.x 9805720

[B5] Campbell OMR Graham WJ Strategies for reducing maternal mortality: getting on with what works Lancet 2006 368 1284 1299 10.1016/S0140-6736(06)69381-1 17027735

[B6] Bloom SS Lippeveld T Wypij D Does antenatal care make a difference to safe delivery? A study in urban Uttar Pradesh, India Health Policy Plan 1999 14 1 38 48 10.1093/heapol/14.1.38 10351468

[B7] Yanagisawa S Oum S Wakai S Determinants of skilled birth attendance in rural Cambodia Trop Med Int Health 2006 11 2 238 51 10.1111/j.1365-3156.2005.01547.x 16451349

[B8] WHO/UNICEF Antenatal care in developing countries: Promises, achievements and missed opportunities. Analysis of trends, levels and differentials 1990-2001 2003 WHO, Geneva

[B9] Stanton C Blanc AK Croft T Choi Y Skilled care at birth in the developing world: progress to date and strategies for expanding coverage J Biosoc Sci 2007 39 109 120 10.1017/S0021932006001271 16522226

[B10] Hill K Thomas K AbouZahr C Walker N Say L Inoue M Suzuki E on behalf of the maternal working group Estimates of maternal mortality worldwide between 1990 and 2005: an assessment of the available data Lancet 2007 370 9595 1311 19 10.1016/S0140-6736(07)61572-4 17933645

[B11] United Nations Children's Fund State of the world's children, 2009: Maternal and Newborn Health 2009 New York. United Nations Children's Fund (UNICEF)

[B12] Thaddeus S Maine D Too far to walk: maternal mortality in context Soc Sci Med 1994 38 8 1091 1110 10.1016/0277-9536(94)90226-7 8042057

[B13] Ensor T Cooper S Overcoming barriers to health service access: influencing the demand side Health Policy Planning 2004 19 2 69 79 10.1093/heapol/czh009 14982885

[B14] JHPIEGO Monitoring birth preparedness and complication readiness. Tool and indicators for maternal and newborn health 2004 Baltimore Maryland, USA

[B15] Carroli G Rooney C Villar J How effective is antenatal care in preventing maternal mortality and serious morbidity? An overview of the evidence Paediatr Perinat Epidemiol 2001 15 suppl 1 1 42 10.1046/j.1365-3016.2001.0150s1001.x 11243499

[B16] National Bureau of Statistics Tanzania 2004-05 Demographic and Health Survey 2006 Ministry of Economic Planning and Development. United Republic of Tanzania, Dar es salaam, Tanzania

[B17] Ministry of Health Focused Antenatal care, Malaria and Syphilis in Pregnancy. Orientation package for Service Providers Reproductive and Child Health Section, Dar es salaam, Tanzania 2002

[B18] National Bureau of Statistics Tanzania Service Provision Assessment Survey 2006 2007 Dar es salaam, Tanzania and Macro International Inc, Calverton, Maryland USA

[B19] von Both C Flebetaa S Makuwani A Mpembeni R Jahn A How much time do health services spend on antenatal care? Implications for the introduction of the focused antenatal care model in Tanzania BMC Pregnancy Childbirth 2006 6 1 22 10.1186/1471-2393-6-22 PMC1557863 16796749

[B20] National Bureau of Statistics Tanzania Population and Housing Census 2002 Dar es salaam, Tanzania

[B21] Keusch GT Wilentz J Kleinman A Stigma and global health: a research agenda Lancet 2006 367 9509 525 527 10.1016/S0140-6736(06)68183-X 16473128

[B22] Ulin PR Robinson ET Tolley EE McNeil ET Collecting qualitative data: the science and the art. In Qualitative Methods. A field guide for applied Research in Sexual and Reproductive Health 2002 Family Health International. Research Triangle Park, NC, USA 69 111

[B23] Glaser BG Basics of grounded theory and analysis 1992 Mill Valley, CA: Sociology Press

[B24] Denzin N Sociological Methods: A sourcebook Aldine Transactions Chicago 2006

[B25] Rosenstock IM Why people use health services The Milbank Quarterly 2005 83 4 1 32 10.1111/j.1468-0009.2005.00425.x

[B26] Nikiema B Beninguisse G Haggerty JL Providing information on pregnancy complications during antenatal visits: unmet educational needs in sub-Saharan Africa Health Policy Planning 2009 24 367 376 10.1093/heapol/czp017 19401360

[B27] Pembe AB Urassa DP Carlstedt A Lindmark G Nystrom L Darj E Rural Tanzanian women's awareness of danger signs of obstetric complications BMC Pregnancy and Childbirth 2008 9 10.1186/1471-2393-9-12 PMC2667432 19323836

[B28] Allen D Managing Motherhood, Managing Risk. Fertility and Dangers in West Central Tanzania 2002 University of Michigan, USA

[B29] Grossmann-Kindall F Filippi V De Koninck M Kanhonou L Giving birth in hospitals in Benin: testimonies of women Reprod Health Matters 2001 9 18 90 98 10.1016/S0968-8080(01)90095-3 11765405

[B30] World Health Organization Pregnancy, childbirth, postpartum and newborn care: a guide for essential practice 2003 WHO, Geneva 26561684

[B31] World Health Organization Working with individuals, families and communities to improve maternal and newborn health Making Pregnancy Safer Initiative. Reproductive Health and Research Unit 2003 WHO, Geneva

[B32] World Health Organization WHO antenatal care randomized trial: manual for the implementation of the new model Geneva (document no WHO/RHR/01.30) 2001

[B33] Onah HE Ikeako LC Iloabachie GC Factors associated with the use of maternity services in Enugu, southeastern Nigeria Soc Sci Med 2006 63 1870 1878 10.1016/j.socscimed.2006.04.019 16766107

[B34] Kyomuhendo GB Low use of maternity services in Uganda: impact on women's status, traditional beliefs and limited resources Reprod Health matters 2003 11 21 16 26 10.1016/S0968-8080(03)02176-1 12800700

[B35] Asuquo EEJ Etuk SJ Duke F Staff attitude as barrier to the utilization of University of Calabar Teaching Hospital for Obstetric care African Journal of Reproductive Health 2000 4 2 69 73 10.2307/3583450

[B36] Kruk ME Paczkowski M Mbaruku G de Pinho H Galea S Women's Preferences for Place of Delivery in Rural Tanzania: A Population-Based Discrete Choice Experiment Am J Public Health 2009 99 1666 1672 10.2105/AJPH.2008.146209 PMC2724466 19608959

